# Efficient deep learning-based automated diagnosis from echocardiography with contrastive self-supervised learning

**DOI:** 10.1038/s43856-024-00538-3

**Published:** 2024-07-06

**Authors:** Gregory Holste, Evangelos K. Oikonomou, Bobak J. Mortazavi, Zhangyang Wang, Rohan Khera

**Affiliations:** 1 Department of Electrical and Computer Engineering, The University of Texas at Austin, Austin, TX USA; 2 Section of Cardiovascular Medicine, Department of Internal Medicine, Yale School of Medicine, New Haven, CT USA; 3 Department of Computer Science & Engineering, Texas A&M University, College Station, TX USA; 4 Center for Outcomes Research and Evaluation, Yale New Haven Hospital, New Haven, CT USA; 5 Section of Biomedical Informatics and Data Science, Yale School of Medicine, New Haven, CT USA; 6 Section of Health Informatics, Department of Biostatistics, Yale School of Public Health, New Haven, CT USA

**Keywords:** Diagnostic markers, Valvular disease, Echocardiography

## Abstract

**Background:**

Advances in self-supervised learning (SSL) have enabled state-of-the-art automated medical image diagnosis from small, labeled datasets. This label efficiency is often desirable, given the difficulty of obtaining expert labels for medical image recognition tasks. However, most efforts toward SSL in medical imaging are not adapted to video-based modalities, such as echocardiography.

**Methods:**

We developed a self-supervised contrastive learning approach, EchoCLR, for echocardiogram videos with the goal of learning strong representations for efficient fine-tuning on downstream cardiac disease diagnosis. EchoCLR pretraining involves (i) contrastive learning, where the model is trained to identify distinct videos of the same patient, and (ii) frame reordering, where the model is trained to predict the correct of video frames after being randomly shuffled.

**Results:**

When fine-tuned on small portions of labeled data, EchoCLR pretraining significantly improves classification performance for left ventricular hypertrophy (LVH) and aortic stenosis (AS) over other transfer learning and SSL approaches across internal and external test sets. When fine-tuning on 10% of available training data (519 studies), an EchoCLR-pretrained model achieves 0.72 AUROC (95% CI: [0.69, 0.75]) on LVH classification, compared to 0.61 AUROC (95% CI: [0.57, 0.64]) with a standard transfer learning approach. Similarly, using 1% of available training data (53 studies), EchoCLR pretraining achieves 0.82 AUROC (95% CI: [0.79, 0.84]) on severe AS classification, compared to 0.61 AUROC (95% CI: [0.58, 0.65]) with transfer learning.

**Conclusions:**

EchoCLR is unique in its ability to learn representations of echocardiogram videos and demonstrates that SSL can enable label-efficient disease classification from small amounts of labeled data.

## Plain language summary

Artificial intelligence (AI) has been used to develop software that can automatically diagnose diseases from medical images. However, these AI models require thousands or millions of examples to properly learn from, which can be very expensive, as diagnosis is often time-consuming and requires clinical expertise. Using a technique called self-supervised learning (SSL), we develop an AI method to effectively diagnose heart disease from as few as 50 instances. Our method, EchoCLR, is designed for echocardiography, a key imaging technique to monitor heart health, and outperforms other methods on disease diagnosis from small amounts of data. This method can advance AI for echocardiography and enable researchers with limited resources to create disease diagnosis models from small medical imaging datasets.

## Abstract (web summary)

Holste et al. present a deep learning method for efficient cardiac disease diagnosis from echocardiography videos. Their method, EchoCLR, uses self-supervised learning to accurately detect heart diseases from a smaller number of labeled examples than existing methods.

## Introduction

Transthoracic echocardiography (TTE) is a cornerstone of cardiovascular care, assessing cardiac structure and function through multi-view ultrasound videos. TTE is ubiquitous due to its non-invasive nature, lack of radiation exposure, real-time imaging capabilities, and increasing portability with handheld ultrasound devices. For these reasons, TTE is often the first cardiac imaging performed and remains the gold standard for many important structural and functional cardiac conditions^1–3^. However, the quality of acquisition can be highly variable, requiring dedicated method development for automated diagnosis from TTE. For this reason, despite the clinical importance of TTE and the rise of deep learning for medical imaging^4^, deep learning techniques have only recently been developed and adapted to detect cardiac diseases from echocardiography^5–10^. While important steps forward, nearly all of these works either (a) examine individual still frames, ignoring the temporal aspect of echocardiography, or (b) rely on standard deep supervised learning for videos, which requires large labeled datasets for training. The latter is particularly burdensome for clinical diagnosis tasks, where acquiring expert labels for thousands of medical videos can be prohibitively time-consuming and expensive^11^. Therefore, we sought to develop a label-efficient self-supervised learning (SSL) approach to learn representations of echocardiogram videos as powerful initializations for downstream disease diagnosis from small, labeled datasets. We validate our proposed approach on the classification of left ventricular hypertrophy (LVH), an abnormal thickening of the left ventricle walls that is measurable from a single TTE video, and aortic stenosis (AS), a disorder causing narrowing of the aortic valve typically diagnosed with complex multi-view and Doppler echocardiography.

The goal of SSL is to learn useful features, or representations, of unlabeled data that can be transferred to a downstream task of interest. In this setting, model development is partitioned into two steps: pretraining (use SSL to learn general features from unlabeled data) and fine-tuning (given a pretrained model, use supervised learning to accomplish a specific task from labeled data). SSL can come in many forms, including pretext tasks (e.g., predict what transformation has been applied to an image or solve a jigsaw puzzle by correctly arranging jumbled image patches)^12,13^, masked image modeling (remove patches of an image, then reconstruct the missing signal from the present patches)^14–16^, and contrastive learning (encourage the model to learn similar features of similar images and dissimilar features of distinct images)^17–19^. These SSL approaches have proven very effective for label-efficient fine-tuning in natural image classification^17,18^, video classification^20,21^, and now even medical image classification and segmentation tasks^22–25^. However, most successful medical applications of SSL operate on 2D data, such as histopathological images and radiographs^24,26,27^. Some recent studies have developed SSL methods for 3D medical image data, though this has typically been applied to computed tomography (CT) and magnetic resonance imaging (MRI), where this third dimension is spatial, not temporal^23,28^. Successful applications of SSL to 3D medical imaging, primarily CT and MRI, are enabled by the high spatial resolution and standardized acquisition of these modalities. Ultrasound, on the other hand, is a noisier modality captured manually by a sonographer with frequent motion during acquisition, producing low-contrast images that are sensitive to transformation. Moreover, echocardiography is highly dependent on the skill of the sonographer, with frequent off-axis images containing distortions and artifacts^29^.

There are two main challenges to applying contrastive SSL to echocardiography: (i) ultrasonography is a low-contrast, noisy imaging modality, and (ii) echocardiograms contain rich temporal information. First, SimCLR^17^, a popular contrastive learning framework, uses extreme image augmentations to produce two views, or randomly transformed copies, of an image; the model is then trained to learn similar feature vectors (representations) of these two copies originating from the same image and dissimilar representations of all other pairs of images. Naively applying such heavy augmentations to ultrasound images will destroy valuable signal, rendering the images useless for diagnosis. To tackle this first issue, we employ multi-instance echocardiography sampling. In a routine TTE study, a patient will often have multiple videos captured from a single echocardiographic view during acquisition; we can then leverage these different videos of a given patient as positive pairs, for which the model will learn to produce similar representations. This forms a challenging contrastive learning task and, critically, removes the need for augmentation to artificially generate two views of a patient, instead using authentically distinct videos. Second, many contrastive learning approaches, including SimCLR, were developed for 2D natural images, failing to model the complex temporal patterns in echocardiogram videos. To address this second issue, we utilize a pretext task of frame reordering. For this, we randomly shuffle the frames of each echocardiogram video clip and train the model to correctly predict the exact order in which the frames have been shuffled. This encourages temporal understanding that we observe to aid downstream severe AS and LVH diagnosis and generate more interpretable visual explanations of these predictions.

Through validation on internal and external testing data, we demonstrate that the proposed method, EchoCLR, significantly outperforms existing transfer learning and SSL methods for both LVH and severe AS classification when trained on a small number of labeled TTE studies (as few as 51). While existing work applies contrastive learning to echocardiography^30–32^, these efforts only operate on individual still frames and do not perform downstream disease classification. To our knowledge, this represents the first effort toward self-supervised contrastive learning of echocardiogram videos for label-efficient cardiac disease diagnosis.

## Methods

### Data curation and deidentification

An initial pull of 12,500 TTE studies conducted at Yale New Haven Hospital from 2016 to 2021 was extracted for this study. This pull consisted of 10,000 studies from 2016 to 2020 to be used for model development and 2500 studies from 2021 to be used for model evaluation. Since severe AS is an uncommon condition^33^, the 10,000 studies from 2016 to 2020 oversampled severe AS by a factor of 50 and non-severe AS by a factor of 5 to ensure a sufficient number of positive examples for reliable model development and validation. The remaining 2500 studies from 2021 were *not* enriched for AS, serving as a temporally distinct external test set. Of the 10,865 studies properly extracted from the database, 9710 studies contained valid pixel data. The resulting 447,653 videos were then deidentified by masking boundary pixels in each frame to remove protected health information, then converted to Audio Video Interleave (AVI) format for fast data loading. The view classification and video preprocessing details below follow those described in our previous work^10^, though the cohort of studies used in this work differs.

This study was approved by the Yale University Institutional Review Board, which waived the need for informed consent as the study represents secondary analysis of existing data (Yale IRB ID #2000029973).

### View Classification

This study leveraged single-view echocardiography from the parasternal long axis (PLAX) view by employing a pretrained TTE view classifier developed by Zhang et al. ^34^ For all 447,653 videos, ten deidentified frames were randomly selected, downsampled to 224 × 224 resolution, and fed into the view classifier. The 10 frame-level predicted view probabilities were then averaged into a single video-level view prediction. While the pretrained view classifier was capable of discriminating variants of the canonical PLAX view (e.g., “PLAX – zoom of the left atrium,” “PLAX – remote,” etc.), we retained videos most confidently classified as “PLAX.”

### Preprocessing

After view classification, the 30,136 PLAX videos from 9173 studies were prepared for deep learning model development. We excluded echocardiograms with low-flow, low-gradient AS given differences in AS severity measures across domains and due to the difficulty of categorizing the severity of this paradoxical form of AS, leaving 29,978 PLAX videos from 9122 studies. All videos then underwent an image processing pipeline that binarized each video frame with a fixed threshold of 200, then masked all pixels outside the convex hull of the largest contour to remove information outside the central image content. After these steps, each video clip was spatially downsampled to 112 × 112 resolution. All videos from studies conducted from 2016 to 2020 were then randomly split at the study level into training (75%), validation (10%), and internal test (15%) sets at the study level. See Table S1 for detailed demographic details of the resulting study cohort.

### Echocardiogram labeling

Echocardiographic measurements and reported diagnoses are reported in accordance with the recommendations of the American Society of Echocardiography (ASE)^1,35,36^. Specifically, the presence of AS severity was determined by the original echocardiographic report and reflected the final severity grade assigned by the interpreting physician. Since severe AS detection was formulated as a binary classification task, all AS designations other than “severe AS” were binned into the “not severe AS” category. LVH was defined based on sex-specific thresholds for the left ventricular mass index, namely >95 g/m^2^ in women and >115 g/m^2^ in men^35^.

### Self-supervised pretraining

**Fig. 1:**
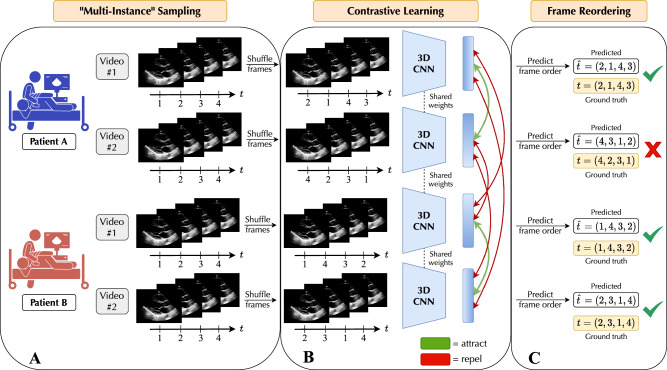
Overview of EchoCLR, a self-supervised learning approach for echocardiography. Unlike standard contrastive learning methods, two *distinct* videos of each patient acquired during a single exam are randomly sampled and deemed positive pairs for multi-instance contrastive learning (**A**). The frames of each video are then randomly shuffled along the temporal axis and fed into a 3D CNN, which learns *similar* representations of distinct videos from the same patient and *dissimilar* representations of videos from different patients (**B**). These video-level representations are then used to directly predict the order of shuffled video frames. This frame reordering pretext task encourages temporal coherence, which we demonstrate to be beneficial for downstream echocardiogram video-based disease classification tasks (**C**). After self-supervised pretraining, the 3D CNN backbone can then be efficiently fine-tuned for cardiac disease classification based on very few labeled echocardiograms. CNN = convolutional neural network.

To learn transferable representations of PLAX echocardiogram videos for downstream cardiac disease classification, we performed self-supervised pretraining on all training set videos. This pretraining step enables the model to learn representations of echocardiograms that are robust to variations in video acquisition and more rapidly adapt to the target fine-tuning task than other initialization approaches. We designed an SSL algorithm specifically catered to echocardiogram videos, EchoCLR, which consists of (i) a multi-instance contrastive learning task and (ii) a frame reordering pretext task (Fig. 1).

We adopted the SimCLR framework^17^ for contrastive learning, which produces two views of an image by sending two copies of the input through a pipeline of random image augmentations, producing view ${\widetilde{x}}_{i}$ and ${\widetilde{x}}_{j}$. An encoder $f()$ is then used to learn representations of each view, ${h}_{i}=f({\widetilde{x}}_{i})$ and ${h}_{j}=f({\widetilde{x}}_{j})$, which are then projected to a lower dimensionality with a projector $g()$. The resulting learned embeddings of each view, ${z}_{i}=g(f({\widetilde{x}}_{i}))$ and ${z}_{j}=g(f({\widetilde{x}}_{j}))$ are then contrasted via the temperature-normalized cross-entropy (NT-Xent) loss, which encourages the model to learn similar representations of views from the same original image (positive pairs) and dissimilar representations of views from all other images (negative pairs) in a given minibatch. This loss is computed via

1 $${\ell}_{i,j}=-={log} \frac{\exp \left(\frac{{sim}\left({z}_{i},{z}_{j}\right)}{\tau }\right)}{{\sum }_{k=1}^{2N}{{\mathbb{1}}}_{\left[i\ne k\right]}\exp \left(\frac{{sim}\left({z}_{i},{z}_{k}\right)}{\tau }\right)},$$

ere ${sim}(\cdot )$ is the cosine similarity and $\tau$ is a hyperparameter referred to as temperature; the final loss is the average ${\ell}_{i,j}$ for all positive pairs $(i,j)$ and $(j,i)$ in the minibatch.

Borrowing language and insights from Azizi et al. ^37^, we form positive pairs between different videos of the same patient (acquired during the same study). This removes the need to synthetically create two different views of a patient by transforming their echocardiogram, instead leveraging the fact that routine TTE studies contain multiple unique PLAX videos. Throughout the paper, this approach is referred to as multi-instance SimCLR (MI-SimCLR). To address the temporal nature of echocardiography, we additionally included a frame reordering pretext task, where we randomly permuted the frames of each input echocardiogram video clip, then trained the model to predict the shuffled order of frames. Similar to the approach of Jiao et al. ^12^, this frame reordering task is treated as a classification problem and was implemented with a fully-connected layer that minimizes the cross-entropy between the known and predicted frame order; specifically, if an input video clip has $K$ frames, then the $K!$ possible permutations of frames served as the targets for classification. The final loss function of EchoCLR is the sum of the contrastive NT-Xent objective and the pretext frame reordering cross-entropy objective.

Self-supervised pretraining was performed on randomly sampled video clips of $K=4$ consecutive frames from all training set echocardiogram videos. Since the number of orderings increases factorially with $K$, this short video clip length was chosen because it provides enough temporal information to model while being computationally efficient and avoiding repeated stages of the cardiac cycle that could challenge frame reordering. The encoder $f()$ was a randomly initialized 3D-ResNet18^38^, and the projector $g()$ projected each 512-dimensional learned representation down to a 128-dimensional representation with a hidden layer of 256 units followed by a ReLU activation, followed by an output layer with 128 units. Each model used the Adam optimizer^39^, a learning rate of 0.1, a batch size of 392 (196 per GPU), and an NT-Xent temperature hyperparameter of 0.5. The following augmentations were applied to each frame within a video clip: random zero padding by up to 8 pixels in each spatial dimension, a random horizontal flip with probability 0.5, and a random rotation within (−10, 10) degrees with probability 0.5. For methods that used multi-instance sampling (MI-SimCLR and EchoCLR), the model was trained for 300 epochs on all unique pairs of different PLAX videos acquired in the same study. Since SimCLR does not use multi-instance sampling, this method was trained for 520 epochs to match the number of optimization steps (or, examples seen) during MI-SimCLR and EchoCLR pretraining for a fair comparison. Each model was pretrained on two NVIDIA RTX 3090 GPUs.

### Supervised fine-tuning

The same 3D-ResNet18 encoder described above was used to classify LVH and severe AS. Three different methods were used to initialize the parameters of this network: an SSL initialization (using one of SimCLR, MI-SimCLR, or EchoCLR), a Kinetics-400 initialization, and a random initialization. The SSL initializations directly used the learned weights of the encoder from the one of the three SSL pretraining approaches described in detail above. The Kinetics-400 initialization represents a standard transfer learning approach for spatiotemporal data, using the weights from a 3D-ResNet18 trained in a supervised fashion on the Kinetics-400 dataset, a large corpus of over 300,000 natural videos for human action classification; these weights are readily available through the *torchvision* API (https://pytorch.org/vision/stable/index.html) provided by PyTorch^40^. To understand the label efficiency of each model initialization method, a training ratio (or, data titration) experiment was performed: for each disease and initialization, a 3D-ResNet18 was initialized with the given method and fine-tuned on 1%, 5%, 10%, 25%, 50%, and 100% of all available training set TTE studies for the given disease. For LVH classification, this involved using 51 (1%), 259 (5%), 519 (10%), 1298 (25%), 2597 (50%), and 5194 (100%) studies for fine-tuning; for severe AS, this involved 53 (1%), 265 (5%), 531 (10%), 1327 (25%), 2655 (50%), and 5311 (100%) studies.

All fine-tuned models were trained on randomly sampled clips of 16 consecutive frames from training set echocardiograms. The same augmentations were used as in the self-supervised pretraining described above. However, when fine-tuning from a Kinetics-400 initialization, video clips were further standardized using the channel-wise means and standard deviations from the Kinetics-400 training dataset, a standard preprocessing step when performing transfer learning. All models were trained for a maximum of 30 epochs with early stopping – specifically, if the validation loss did not improve for 5 consecutive epochs, training was terminated, and the weights from the epoch with minimum validation loss were used for final evaluation. All fine-tuned models were trained on a single NVIDIA RTX 3090 GPU with the Adam optimizer and a batch size of 88 to maximize GPU utilization. When fine-tuning on more than 10% of available training data, the Kinetics-pretrained and randomly initialized models used a learning rate of $1\times {10}^{-4}$, while the SSL-pretrained models used a learning rate of 0.1. When fine-tuning on 10% or less of available data, the Kinetics-pretrained and randomly initialized models used a learning rate of $1\times {10}^{-4}$, $5\times {10}^{-5}$, or $1\times {10}^{-5}$, while the SSL-pretrained models used a learning rate of 0.1, 0.05, or 0.001; this was done due to the stochasticity introduced by training on a very small subset of data, and the single learning rate with minimum validation loss at the early stopping-determined checkpoint was ultimately selected.

### Model interpretability

To assess the interpretability of EchoCLR and rule out reliance on spurious shortcuts that are unrelated to diagnostic signal^41^, a saliency map analysis was conducted for severe AS diagnosis. After model training, the Grad-CAM method^42^ was used to obtain saliency heatmaps, providing a visual explanation of which parts of the input video contribute most to its disease prediction. Specifically, heatmaps were generated by applying Grad-CAM to a clip of the first 32 frames of an echo, using the last convolution block of the 3D-ResNet18 to generate a $7\times 7\times 4$ (height $\times$ width $\times$ time) heatmap displaying roughly where the model is attending to over the spatial and temporal dimensions. The Grad-CAM output was interpolated to the original input dimension of $112\times 112\times 32$ with the scipy^43^ *zoom* function, providing a spatiotemporal 3D saliency map. However, to generate a single 2D heatmap for a given video clip, the pixelwise maximum along the temporal dimension was taken to capture the most salient regions for severe AS diagnosis across all timepoints.

### Model performance evaluation

Since severe AS and LVH labels describe each TTE study, but the model is trained on multiple echocardiogram video clips from the same study, video-level predictions of disease presence are averaged at inference time to obtain a single study-level prediction. All fine-tuned severe AS and LVH classification models were evaluated by AUROC and area under the precision-recall curve (AUPR), which can be useful when evaluating a classifier’s performance in the presence of imbalanced data.

### Statistics and reproducibility

All 95% confidence intervals for model evaluation metrics were computed by bootstrapping. At the study level, 10,000 bootstrap samples (samples with replacement of the same size as the evaluation set) were drawn, metrics were computed on this bootstrapped set, and nonparametric confidence intervals were generated with the percentile method^44^. All p-values were computed with a Python implementation of the “bootstrap” method described in the documentation for the roc.test function (https://cran.r-project.org/web/packages/pROC/pROC.pdf) in the pROC library^45^. Significance tests were one-sided—the alternative hypothesis being that the EchoCLR-pretrained model’s AUROC exceeded the other model’s AUROC—with a significance level 0.05. All experiments can be reproduced via our open-source code repository at https://github.com/CarDS-Yale/EchoCLR.

### Reporting summary

Further information on research design is available in the Nature Portfolio Reporting Summary linked to this article.

## Results

### Study cohort description

This study included patients who underwent transthoracic echocardiograms (TTE) between the years 2016 and 2021 at the Yale New Haven Hospital, with LVH and severe AS labels determined from cardiologist interpretation. A sample of 10,000 studies from 2016 to 2020 was extracted for model development and internal testing, while a sample of 2500 studies from 2021 was extracted to serve as a temporally distinct external test set. To obtain videos solely from the parasternal long axis (PLAX) view—the first and most common view obtained in TTE—a pretrained automatic view classifier was employed^34^. Following view classification, the resulting PLAX videos underwent deidentification by using an image processing pipeline to mask pixels outside of the central image content. The resulting 2016–2020 cohort consisted of 23,448 videos from 7082 TTE studies, while the temporally distinct 2021 cohort consisted of 6530 videos from 2,040 studies. While AS labels were present for all studies, LVH labels could only be determined for a subset of 22,952 videos from 6931 studies in the 2016–2020 cohort and 6379 videos from 1995 studies in the 2021 cohort. Demographic information can be found in Table 1 and full data curation and preprocessing details can be found in the Methods.

**Table 1:** Description of study cohort

		Overall	Training	Validation	InternalTesting	External Testing
**TTE Studies**, ***n***		9122	5311	708	1063	2040
**Age (years), mean (SD)**		69.1 (16.0)	70.2 (15.8)	70.1 (15.6)	69.8 (15.8)	65.7 (16.4)
**Gender, n (%)**	**Female**	4467 (49.0)	2600 (49.0)	349 (49.3)	521 (49.0)	997 (48.9)
	**Male**	4655 (51.0)	2711 (51.0)	359 (50.7)	542 (51.0)	1043 (51.1)
**Race, n (%)**	**Asian**	120 (1.3)	60 (1.1)	6 (0.8)	14 (1.3)	40 (2.0)
	**African American**	836 (9.2)	468 (8.8)	68 (9.6)	96 (9.0)	204 (10.0)
	**Other**	498 (5.5)	272 (5.1)	35 (4.9)	56 (5.3)	135 (6.6)
	**Unknown**	929 (10.2)	487 (9.2)	80 (11.3)	114 (10.7)	248 (12.2)
	**White/Caucasian**	6739 (73.9)	4024 (75.8)	519 (73.3)	783 (73.7)	1413 (69.3)
**BMI (kg/m^2), mean (SD)**		29.5 (16.3)	29.4 (19.6)	30.1 (16.7)	29.4 (8.2)	29.4 (7.3)
**Severe aortic valve stenosis,** ***n*** **(%)**		1609 (17.6)	1183 (22.3)	160 (22.6)	246 (23.1)	20 (1.0)
**Left ventricular hypertrophy,** ***n*** **(%)**		2280 (25.5)	1398 (26.9)	199 (28.6)	302 (29.0)	381 (19.1)

Descriptive statistics of demographics and label prevalence for each set of the study cohort. Percentages are valid percentages calculated for studies with available information. *BMI* body mass index, *SD* standard deviation, *TTE* transthoracic echocardiography.

### Label-efficient LVH classification

To evaluate label efficiency, we compared three different initialization strategies (random weights, Kinetics-400 weights, and EchoCLR-pretrained weights) and fine-tuned each model on increasing amounts of training data, ranging from 1% to 100% of available data. When using all available training data (5194 studies), EchoCLR pretraining and Kinetics-400 pretraining, representing a standard video-based transfer learning approach, classified LVH comparably. With all training data, an EchoCLR-pretrained model reached 0.795 AUROC (95% CI: [0.770, 0.819]) on the internal test set and 0.804 AUROC (95% CI: [0.783, 0.824]) on the external test set, compared to 0.807 AUROC (95% CI: [0.783, 0.830], *P* = 0.818) in internal testing and 0.806 AUROC (95% CI: [0.786, 0.827], *P* = 0.599) in external testing with Kinetics-400 pretraining.

**Fig. 2:**
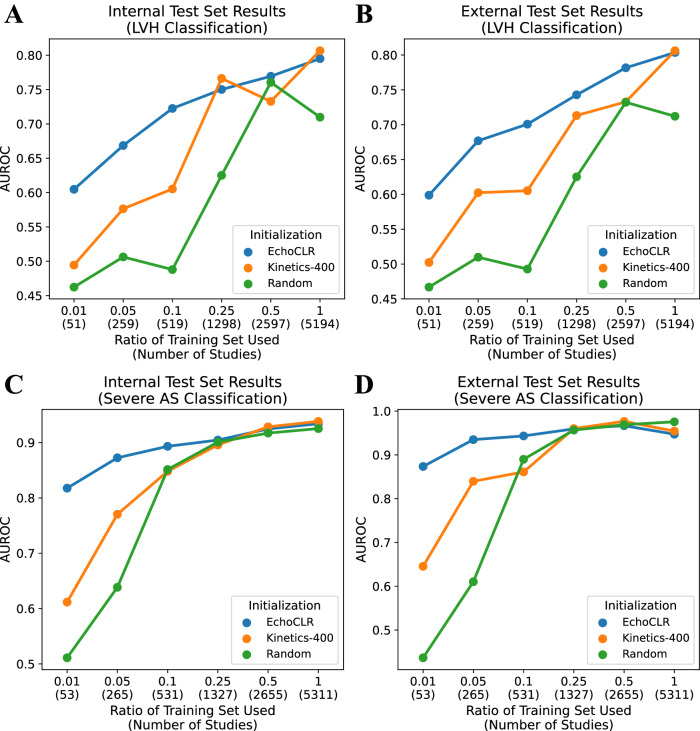
Classification performance on different amounts of training data. AUROC for LVH classification on the internal (**A**) and external test set (**B**) and severe AS classification on the internal (**C**) and external test set (**D**) for a randomly initialized, Kinetics-400-pretrained, and EchoCLR-pretrained model when fine-tuned on different amounts of labeled training data. AS aortic stenosis; AUROC area under the receiver operating characteristic curve, LVH left ventricular hypertrophy.

However, when using less than 25% of training data for fine-tuning (<1000 studies), EchoCLR pretraining consistently outperformed other initialization methods on downstream LVH classification, as measured by AUROC in internal and external test sets (Fig. 2A, B). For example, when using only 10% of training data for fine-tuning (519 studies), an EchoCLR-pretrained model reached 0.723 AUROC (95% CI: [0.693, 0.751]), significantly outperforming the Kinetics-400-pretrained model (0.605 AUROC, 95% CI: [0.574, 0.636], *P* < 0.001) and randomly initialized model (0.488 AUROC, 95% CI: [0.456, 0.521], *P* < 0.001); in the external test set, the EchoCLR-pretrained model reached 0.701 AUROC (95% CI: [0.676, 0.725]), again outperforming the Kinetics-400-pretrained model (0.605 AUROC, 95% CI: [0.578, 0.632], *P* < 0.001) and randomly initialized model (0.493 AUROC, 95% CI: [0.465, 0.520], *P* < 0.001). Overall, EchoCLR pretraining significantly improved upon Kinetics-400 transfer learning on 1%, 5%, 10%, and 50% training ratios in the internal test set and all training ratios except 100% in the external test set. These same trends were also reflected in performance by AUPR (Fig. S1A, S1B), and full results for LVH classification can be found in Table S2.

### Label-efficient severe AS classification

Aligned with the patterns observed in LVH classification, EchoCLR and Kinetics-400 initializations performed similarly for downstream severe AS classification. Leveraging all available fine-tuning data (5,311 studies), EchoCLR pretraining achieved 0.934 AUROC (95% CI: [0.920, 0.947]) on the internal test set and 0.947 AUROC (95% CI: [0.884, 0.988]) on the external test set, while Kinetics-400 pretraining reached 0.938 AUROC (95% CI: [0.925, 0.951], *P* = 0.774) in internal testing and 0.954 (95% CI: [0.921, 0.983], *P* = 0.615) in external testing.

Once again, however, EchoCLR pretraining improved severe AS classification when leveraging relatively small portions of labeled data (<1000 labeled TTE studies) (Fig. 2C, D). For example, when fine-tuned on only 1% of training data (53 studies), our EchoCLR-pretrained model reached 0.818 AUROC (95% CI: [0.793, 0.840]) in the internal test set, significantly outperforming a Kinetics-400-pretrained model (0.612 AUROC, 95% CI: [0.577, 0.647], *P* < 0.001) and randomly initialized model (0.511 AUROC, 95% CI: [0.477, 0.545], *P* < 0.001). Further, in external testing, the EchoCLR-pretrained model reached 0.874 AUROC (95% CI: [0.820, 0.922]), outperforming the Kinetics-400-pretrained model (0.645 AUROC, 95% CI: [0.525, 0.761], *P* < 0.001) and randomly initialized model (0.437 AUROC, 95% CI: [0.338, 0.535], *P* < 0.001). Overall, EchoCLR pretraining significantly outperformed the standard transfer learning approach of Kinetics-400 pretraining on 1%, 5%, and 10% training ratios in both the internal and external test sets. In other words, once fine-tuning on at least 25% of training data (>1000 studies), all initialization methods were comparable in terms of downstream severe AS classification performance. Consistent trends were also observed with respect to AUPR (Fig. S1C, S1D); see Table S3 for full results.

### The impact of multi-instance sampling and frame reordering on EchoCLR

**Fig. 3:**
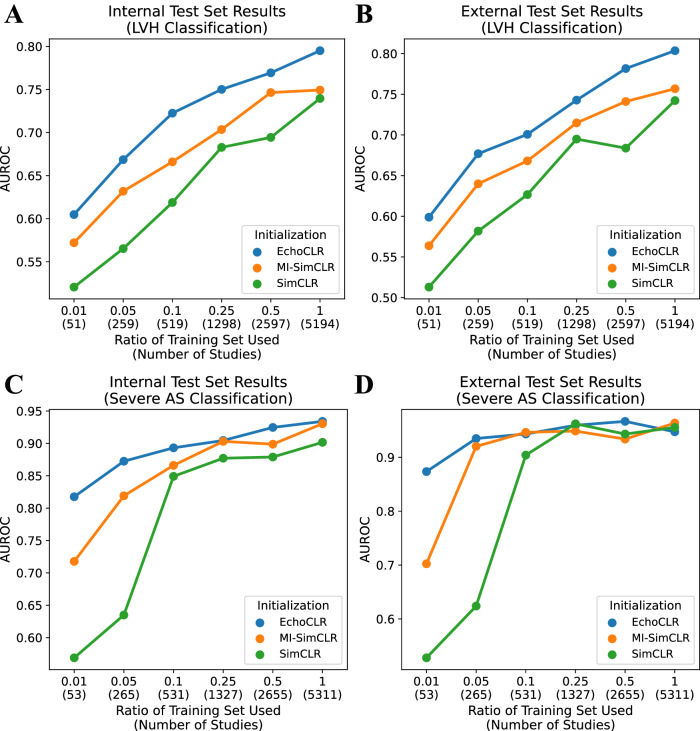
Comparing contrastive learning methods when fine-tuned on different amounts of training data. AUROC for LVH classification on the internal (**A**) and external test set (**B**) and severe AS classification on the internal (**C**) and external test set (**D**) for a SimCLR-pretrained, MI-SimCLR-pretrained, and EchoCLR-pretrained model when fine-tuned on different amounts of labeled training data. AS aortic stenosis AUROC area under the receiver operating characteristic curve, LVH left ventricular hypertrophy, MI-SimCLR multi-instance SimCLR.

To demonstrate the additive impact of each component of EchoCLR, we compared the downstream classification performance of (i) EchoCLR, (ii) EchoCLR without frame reordering (MI-SimCLR), and (iii) EchoCLR with neither frame reordering nor multi-instance sampling (SimCLR). In this ablation study, EchoCLR pretraining considerably improved both LVH and severe AS classification over other self-supervised pretraining approaches when fine-tuning on the vast majority of training set ratios. Additionally, the proposed multi-instance echocardiography sampling and frame reordering provided complementary improvements to downstream disease diagnosis (Fig. 3).

To illustrate this, when using just 1% of data for severe AS fine-tuning (53 studies), the EchoCLR-pretrained model reached 0.818 AUROC (95% CI: [0.793, 0.840]), significantly outperforming an MI-SimCLR-pretrained model (0.718 AUROC, 95% CI: [0.685, 0.751], *P* < 0.001) and SimCLR-pretrained model (0.569 AUROC, 95% CI: [0.534, 0.604], *P* < 0.001); in the external test set, the EchoCLR-pretrained model achieved 0.874 AUROC (95% CI: [0.820, 0.922]), outperforming both the MI-SimCLR-pretrained model (0.702 AUROC, 95% CI: [0.610, 0.795], *P* < 0.001) and SimCLR-pretrained model (0.528 AUROC, 95% CI: [0.427, 0.628], *P* < 0.001). When using 10% of training data for LVH fine-tuning (519 studies), the EchoCLR-pretrained model achieved 0.723 AUROC (95% CI: [0.693, 0.751]), again outperforming the MI-SimCLR pretraining (0.666 AUROC, 95% CI: [0.636, 0.696], *P* < 0.001) and SimCLR pretraining (0.619 AUROC, 95% CI: [0.587, 0.650]. *P* < 0.001). Similar trends were observed in performance as measured by AUPR for both LVH (Table S2) and severe AS (Table S3) classification in internal and external testing (Fig. S2).

### Improved interpretability with EchoCLR

**Fig. 4:**
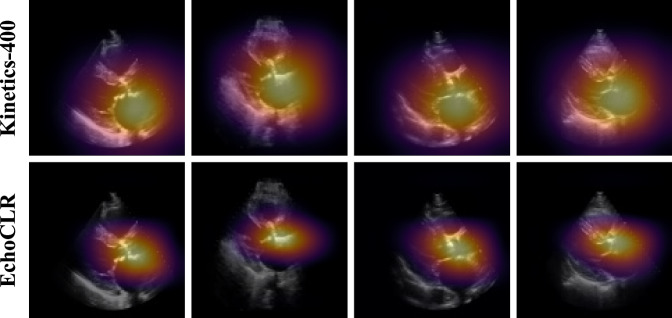
Interpretability analysis of EchoCLR. Saliency map visualizations for the four most confident severe AS predictions as determined by the Kinetics-400-pretrained model when fine-tuned on all training data. For each video, attention maps are presented for the Kinetics-400-pretrained model **(top row)** and the EchoCLR-pretrained model **(bottom row)**. The EchoCLR-pretrained model more closely attends to the aortic valve and annulus than a standard transfer learning model when predicting severe aortic stenosis. While saliency maps were computed for entire video clips, only the first frame of each video is displayed above in each column. Saliency maps were obtained by applying Grad-CAM^42^ to the first 32 frames of each video, then computing the pixel-wise maximum along the temporal axis to obtain a single 2D heatmap.

In addition to evaluating quantitative disease classification performance, we perform a comparative interpretability analysis of EchoCLR vs. a standard transfer learning approach (Kinetics-400 pretraining). To ensure that clinically relevant regions of the patient’s imaging contribute to the model’s predictions, saliency maps were generated for the four most confident severe AS predictions for the Kinetics-400-pretrained and EchoCLR-pretrained model (utilizing all available training data) with GradCAM^42^. These saliency maps provide a visual explanation of which image locations contribute most the model’s disease prediction. When predicting the presence of severe AS, the EchoCLR-pretrained model more closely attended to clinically relevant regions—the aortic valve and annulus—than the Kinetics-400-pretrained model (Fig. 4). In contrast, the Kinetics-400-pretrained model’s heatmaps were far more diffuse, often capturing the entire left atrium, which should not necessarily be relevant to severe AS diagnosis. Refer to the Methods for full details on saliency map generation and visualization.

## Discussion

We developed EchoCLR, the first (to our knowledge) spatiotemporal self-supervised contrastive learning method specifically catered to echocardiography, a key video-based medical imaging modality. Through extensive internal and external validation, we showed that EchoCLR pretraining improved downstream classification for a diverse set of complex classification tasks by 5–40% when fine-tuned on small amounts of labeled data (<1000 TTE studies) compared to other deep learning initialization approaches. In the low-data regime, EchoCLR pretraining consistently outperformed randomly initializing weights and the standard video-based transfer learning with Kinetics-400-pretrained weights on both LVH and severe AS classification. These results demonstrate the label efficiency of in-domain self-supervised pretraining on echocardiograms with EchoCLR, enabling accurate disease classification from small labeled echocardiographic datasets.

Further, our ablation study on EchoCLR demonstrates that the label efficiency does not merely come from pretraining on echocardiograms, but rather the modality-informed modifications proposed in EchoCLR: multi-instance echocardiography sampling and frame reordering. It was hypothesized that (i) using distinct videos of the same patient as positive pairs for contrastive learning would remove the need for heavy augmentation and enable more effective representation learning, and (ii) using a frame reordering pretext task would enforce temporal coherence that existing methods like SimCLR could not. These hypotheses were supported by the observation that MI-SimCLR (SimCLR with multi-instance sampling) improved upon SimCLR (no multi-instance sampling), and EchoCLR (MI-SimCLR with frame reordering) improved upon MI-SimCLR (no frame reordering) across independent downstream disease diagnosis tasks over nearly all fine-tuning ratios in both internal and external testing.

Despite the benefits of self-supervised contrastive pretraining when fine-tuning on small amounts of labeled data, our experiments reveal a saturation point of approximately 25% of training data (>1000 TTE studies), after which all initialization methods were usually comparable (no significant difference in downstream classification performance). Other works on self-supervised learning observe this behavior^46,47^, which may be explained by the fact that while in-domain SSL may enable more rapid adaptation to a downstream task, a sufficient number of labeled examples may provide enough signal for any reasonable initialization to reach a similar minimum in the loss landscape. However, several studies on 2D medical image-based applications of SSL have found that SSL pretraining can even outperform fully supervised approaches in the presence of large amounts of labeled data^15,22,48^.

Future work may explore ways to combine contrastive pretraining with other types of SSL, such as generative modeling^49,50^ and masked autoencoding (MAE)^15,48,51^. While this study used a 3D-ResNet18 encoder, EchoCLR is compatible with any vision encoder capable of modeling video data. Spatiotemporal vision Transformers^52–54^ could serve as an alternative architecture of choice for future SSL efforts in echocardiography, particularly for MAE pretraining given their patch-based representations of visual data. While this study was limited to single-view echocardiography—to isolate the effect of EchoCLR on two key diagnostic tasks from one of the most widely acquired echocardiographic views—developing an SSL method for multi-view echocardiography would allow for even richer representation learning. Additionally, these SSL approaches can further be applied to multimodal data, learning holistic joint representations across modalities, such as between paired echocardiograms and other diagnostic cardiovascular assessments. The chosen applications of LVH and AS, relatively common diseases, allowed us to evaluate performance against the best-case scenario of abundant training data in simulated scenarios where labeled data was infrequent. Future work may examine the applicability of SSL pretraining for rare (or, few-shot) disease detection, where labeled examples are extremely scarce.

Methods like EchoCLR can be used in any clinical setting where it is difficult to acquire large-scale, expert-labeled medical imaging datasets and may have the potential to accelerate deep learning for low-prevalence disease detection. In addition, our open-source implementation should enable practitioners with access to relatively small echocardiography datasets to perform EchoCLR pretraining on their own data for improved label efficiency.

### Supplementary information

Supplementary Materials

Description of Additional Supplementary Files

Supplementary Data 1

Reporting Summary

## Data Availability

The data used in this study are not available for public sharing given the restrictions in our institutional review board approval. Deidentified test data may be made available to researchers under a data use agreement after publication in a peer-reviewed journal. Source data to reproduce Fig. 2 and Fig. 3 can be found in Supplementary Data 1.

## Code availability

The code repository for this work can be found at https://github.com/CarDS-Yale/EchoCLR^55^.
