# Supplementary material for: Efficient deep learning-based automated diagnosis from echocardiography with contrastive self-supervised learning
Source: Commun Med (Lond). 2024 Jul 6;4:133. doi: 10.1038/s43856-024-00538-3 (PMC11227494; doi:10.1038/s43856-024-00538-3)
Supplement: Supplementary file 1 — Supplementary Materials [file 43856_2024_538_MOESM1_ESM.pdf]

Supplementary Materials for "Efficient deep learning-based automated diagnosis from echocardiography with contrastive self-supervised learning"

Gregory Holste[1], Evangelos K. Oikonomou[2], Bobak J. Mortazavi[3], Zhangyang Wang[1], Rohan Khera[2]

[1] Department of Electrical and Computer Engineering, The University of Texas at Austin, Austin, TX, USA
[2] Section of Cardiovascular Medicine, Department of Internal Medicine, Yale School of Medicine, New Haven, CT, USA
[3] Department of Computer Science & Engineering, Texas A&M University, College Station, TX, USA

Address for correspondence:
Rohan Khera, MD, MS
195 Church St, 6th Floor, New Haven, CT 06510
203-764-5885; rohan.khera@yale.edu; @rohan_khera

Table S1 | Detailed description of study cohort

		Overall	Training	Validation	Internal Testing	External Testing
**TTE Studies, n**		9,122	5,311	708	1,063	2,040
**Age (years), mean (SD)**		69.1 (16.0)	70.2 (15.8)	70.1 (15.6)	69.8 (15.8)	65.7 (16.4)
**Gender, n (%)**	**Female**	4,467 (49.0)	2,600 (49.0)	349 (49.3)	521 (49.0)	997 (48.9)
	**Male**	4,655 (51.0)	2,711 (51.0)	359 (50.7)	542 (51.0)	1,043 (51.1)
**Race, n (%)**	**Asian**	120 (1.3)	60 (1.1)	6 (0.8)	14 (1.3)	40 (2.0)
	**African American**	836 (9.2)	468 (8.8)	68 (9.6)	96 (9.0)	204 (10.0)
	**Other**	498 (5.5)	272 (5.1)	35 (4.9)	56 (5.3)	135 (6.6)
	**Unknown**	929 (10.2)	487 (9.2)	80 (11.3)	114 (10.7)	248 (12.2)
	**White/Caucasian**	6,739 (73.9)	4,024 (75.8)	519 (73.3)	783 (73.7)	1,413 (69.3)
**Ethnicity, n (%)**	**Hispanic or Latino**	633 (6.9)	344 (6.5)	51 (7.2)	80 (7.5)	158 (7.7)
	**Non-Hispanic**	7,266 (79.7)	4,295 (80.9)	562 (79.4)	848 (79.8)	1,561 (76.5)
	**Unknown**	1,223 (13.4)	672 (12.7)	95 (13.4)	135 (12.7)	321 (15.7)
**BMI (kg/m^2), mean (SD)**		29.5 (16.3)	29.4 (19.6)	30.1 (16.7)	29.4 (8.2)	29.4 (7.3)
**EF (%), mean (SD)**		58.9 (10.6)	59.0 (10.8)	59.2 (10.7)	58.6 (10.8)	59.0 (10.1)
**Aortic Valve Stenosis, n (%)**	**Sclerosis without Stenosis**	906 (9.9)	471 (8.9)	62 (8.8)	90 (8.5)	283 (13.9)
	**Mild**	962 (10.5)	668 (12.6)	79 (11.2)	132 (12.4)	83 (4.1)
	**Moderate**	634 (7.0)	422 (7.9)	80 (11.3)	73 (6.9)	59 (2.9)
	**Severe**	1,609 (17.6)	1,183 (22.3)	160 (22.6)	246 (23.1)	20 (1.0)
**Left Ventricular Hypertrophy, n (%)**		2,280 (25.5)	1,398 (26.9)	199 (28.6)	302 (29.0)	381 (19.1)
**Aortic Valve Peak Velocity (m/s), mean (SD)**		2.2 (1.2)	2.4 (1.3)	2.5 (1.3)	2.4 (1.3)	1.6 (0.6)
**Interventricular Septum Diastole (cm), mean (SD)**		1.1 (0.2)	1.1 (0.2)	1.1 (0.2)	1.1 (0.2)	1.0 (0.2)
**Left Ventricular Posterior Wall Diastole (cm), mean (SD)**		1.0 (0.2)	1.0 (0.2)	1.1 (0.2)	1.1 (0.2)	1.0 (0.2)
**Left Ventricular Internal Diameter Diastole (cm), mean (SD)**		4.6 (0.7)	4.6 (0.7)	4.6 (0.7)	4.6 (0.7)	4.6 (0.7)
**Left Ventricular Internal Diameter Systole (cm), mean (SD)**		3.1 (0.7)	3.1 (0.7)	3.1 (0.7)	3.1 (0.8)	3.1 (0.7)
**Left Ventricular Mass (g), mean (SD)**		175.0 (64.2)	176.5 (63.2)	178.6 (67.7)	180.7 (69.4)	167.0 (61.9)
**Left Ventricular Mass Index (g/m^2), mean (SD)**		91.0 (29.6)	92.1 (29.4)	92.7 (31.0)	93.8 (32.0)	85.9 (27.9)

Descriptive statistics of demographics and label prevalence for each set of the study cohort. Percentages are “valid percentages” calculated for studies with available information. BMI = body mass index; SD = standard deviation; TTE = transthoracic echocardiography.

Table S2 | Detailed LVH classification results

		AUROC Results on Internal Test Set				
		**Echo-CLR**	**Kinetics-400**	**Random**	**MI-SimCLR**	**SimCLR**
**Train Ratio**	**0.01**	0.605 (0.572, 0.637)	0.494 (0.460, 0.528)	0.462 (0.429, 0.494)	0.572 (0.540, 0.603)	0.521 (0.489, 0.552)
	**0.05**	0.669 (0.638, 0.698)	0.576 (0.545, 0.607)	0.506 (0.475, 0.538)	0.632 (0.600, 0.663)	0.565 (0.532, 0.597)
	**0.10**	0.723 (0.693, 0.751)	0.605 (0.574, 0.636)	0.488 (0.456, 0.521)	0.666 (0.636, 0.696)	0.619 (0.587, 0.650)
	**0.25**	0.750 (0.723, 0.777)	0.766 (0.740, 0.792)	0.625 (0.594, 0.656)	0.704 (0.674, 0.732)	0.683 (0.653, 0.713)
	**0.50**	0.769 (0.743, 0.794)	0.733 (0.705, 0.761)	0.761 (0.733, 0.787)	0.746 (0.719, 0.774)	0.694 (0.664, 0.724)
	**1.00**	0.795 (0.770, 0.819)	0.807 (0.783, 0.830)	0.710 (0.683, 0.738)	0.749 (0.722, 0.776)	0.740 (0.713, 0.766)
		AUPR Results on Internal Test Set				
		**Echo-CLR**	**Kinetics-400**	**Random**	**MI-SimCLR**	**SimCLR**
**Train Ratio**	**0.01**	0.371 (0.341, 0.407)	0.311 (0.284, 0.345)	0.266 (0.250, 0.288)	0.339 (0.312, 0.372)	0.299 (0.278, 0.325)
	**0.05**	0.412 (0.382, 0.448)	0.340 (0.315, 0.371)	0.292 (0.272, 0.319)	0.410 (0.376, 0.451)	0.346 (0.318, 0.380)
	**0.10**	0.489 (0.449, 0.534)	0.381 (0.349, 0.418)	0.288 (0.267, 0.315)	0.427 (0.393, 0.467)	0.388 (0.356, 0.427)
	**0.25**	0.561 (0.518, 0.605)	0.554 (0.512, 0.602)	0.393 (0.361, 0.432)	0.464 (0.427, 0.506)	0.458 (0.419, 0.503)
	**0.50**	0.564 (0.522, 0.608)	0.509 (0.468, 0.556)	0.554 (0.511, 0.600)	0.541 (0.501, 0.589)	0.504 (0.462, 0.547)
	**1.00**	0.615 (0.573, 0.656)	0.607 (0.564, 0.655)	0.463 (0.426, 0.505)	0.542 (0.500, 0.588)	0.519 (0.477, 0.565)
		AUROC Results on External Test Set				
		**Echo-CLR**	**Kinetics-400**	**Random**	**MI-SimCLR**	**SimCLR**
**Train Ratio**	**0.01**	0.599 (0.573, 0.625)	0.502 (0.476, 0.531)	0.467 (0.441, 0.493)	0.564 (0.537, 0.592)	0.513 (0.486, 0.540)
	**0.05**	0.677 (0.651, 0.702)	0.602 (0.575, 0.630)	0.510 (0.482, 0.537)	0.640 (0.613, 0.667)	0.582 (0.555, 0.608)
	**0.10**	0.701 (0.676, 0.725)	0.605 (0.578, 0.632)	0.493 (0.465, 0.520)	0.668 (0.642, 0.695)	0.627 (0.601, 0.652)
	**0.25**	0.743 (0.720, 0.766)	0.713 (0.689, 0.737)	0.625 (0.598, 0.652)	0.715 (0.691, 0.739)	0.695 (0.671, 0.720)
	**0.50**	0.782 (0.761, 0.803)	0.733 (0.710, 0.756)	0.732 (0.708, 0.756)	0.741 (0.718, 0.765)	0.684 (0.659, 0.709)
	**1.00**	0.804 (0.783, 0.824)	0.806 (0.786, 0.827)	0.712 (0.688, 0.737)	0.757 (0.735, 0.779)	0.742 (0.719, 0.766)
		AUPR Results on External Test Set				
		**Echo-CLR**	**Kinetics-400**	**Random**	**MI-SimCLR**	**SimCLR**
**Train Ratio**	**0.01**	0.266 (0.242, 0.292)	0.192 (0.180, 0.209)	0.171 (0.162, 0.183)	0.234 (0.215, 0.257)	0.203 (0.188, 0.222)
	**0.05**	0.327 (0.300, 0.360)	0.274 (0.250, 0.303)	0.202 (0.186, 0.223)	0.313 (0.284, 0.347)	0.243 (0.224, 0.270)
	**0.10**	0.349 (0.318, 0.383)	0.277 (0.251, 0.308)	0.192 (0.178, 0.211)	0.337 (0.306, 0.372)	0.273 (0.251, 0.301)
	**0.25**	0.415 (0.379, 0.455)	0.403 (0.368, 0.440)	0.303 (0.275, 0.337)	0.373 (0.340, 0.408)	0.369 (0.335, 0.407)
	**0.50**	0.465 (0.429, 0.505)	0.436 (0.400, 0.474)	0.430 (0.393, 0.473)	0.432 (0.395, 0.470)	0.367 (0.334, 0.403)
	**1.00**	0.517 (0.478, 0.559)	0.520 (0.483, 0.561)	0.412 (0.376, 0.450)	0.429 (0.393, 0.468)	0.442 (0.404, 0.483)

LVH classification results, as measured by AUROC and AUPR, for all fine-tuning ratios on both the internal and external test sets. "Train ratio" refers to the proportion of the available training data used for fine-tuning after initializing the model with the method specific by each column title. Values in parentheses represent 95% confidence intervals determined by bootstrapping the test set. AUPR = are under the precision-recall curve; AUROC = area under the receiver operating characteristic curve; LVH = left ventricular hypertrophy; MI-SimCLR = multi-instance SimCLR.

Table S3 | Detailed severe AS classification results

		AUROC Results on Internal Test Set				
		**Echo-CLR**	**Kinetics-400**	**Random**	**MI-SimCLR**	**SimCLR**
**Train Ratio**	**0.01**	0.818 (0.793, 0.840)	0.612 (0.577, 0.647)	0.511 (0.477, 0.545)	0.718 (0.685, 0.751)	0.569 (0.534, 0.604)
	**0.05**	0.872 (0.853, 0.891)	0.770 (0.744, 0.796)	0.638 (0.605, 0.671)	0.819 (0.794, 0.843)	0.635 (0.601, 0.669)
	**0.10**	0.893 (0.876, 0.910)	0.848 (0.827, 0.869)	0.851 (0.831, 0.871)	0.866 (0.846, 0.886)	0.849 (0.828, 0.870)
	**0.25**	0.904 (0.888, 0.920)	0.896 (0.878, 0.912)	0.901 (0.885, 0.916)	0.903 (0.888, 0.918)	0.877 (0.858, 0.895)
	**0.50**	0.925 (0.911, 0.938)	0.928 (0.915, 0.941)	0.917 (0.903, 0.931)	0.899 (0.883, 0.915)	0.879 (0.860, 0.897)
	**1.00**	0.934 (0.920, 0.947)	0.938 (0.925, 0.951)	0.925 (0.912, 0.938)	0.930 (0.917, 0.943)	0.902 (0.884, 0.918)
		AUPR Results on Internal Test Set				
		**Echo-CLR**	**Kinetics-400**	**Random**	**MI-SimCLR**	**SimCLR**
**Train Ratio**	**0.01**	0.519 (0.475, 0.572)	0.340 (0.304, 0.385)	0.230 (0.213, 0.251)	0.482 (0.434, 0.534)	0.277 (0.253, 0.312)
	**0.05**	0.628 (0.580, 0.679)	0.471 (0.428, 0.522)	0.345 (0.309, 0.387)	0.533 (0.488, 0.582)	0.340 (0.305, 0.380)
	**0.10**	0.666 (0.619, 0.717)	0.597 (0.548, 0.647)	0.612 (0.567, 0.660)	0.603 (0.555, 0.656)	0.575 (0.528, 0.625)
	**0.25**	0.687 (0.641, 0.740)	0.691 (0.645, 0.738)	0.694 (0.650, 0.739)	0.683 (0.638, 0.729)	0.666 (0.622, 0.709)
	**0.50**	0.766 (0.725, 0.808)	0.769 (0.731, 0.809)	0.735 (0.691, 0.779)	0.684 (0.638, 0.733)	0.651 (0.605, 0.697)
	**1.00**	0.818 (0.784, 0.852)	0.816 (0.780, 0.853)	0.749 (0.704, 0.795)	0.769 (0.727, 0.812)	0.713 (0.670, 0.757)
		AUROC Results on External Test Set				
		**Echo-CLR**	**Kinetics-400**	**Random**	**MI-SimCLR**	**SimCLR**
**Train Ratio**	**0.01**	0.874 (0.820, 0.922)	0.645 (0.525, 0.761)	0.437 (0.338, 0.535)	0.702 (0.610, 0.795)	0.528 (0.427, 0.628)
	**0.05**	0.935 (0.898, 0.966)	0.840 (0.760, 0.914)	0.610 (0.487, 0.730)	0.920 (0.871, 0.962)	0.624 (0.507, 0.735)
	**0.10**	0.943 (0.903, 0.973)	0.861 (0.788, 0.925)	0.890 (0.826, 0.943)	0.946 (0.925, 0.966)	0.904 (0.863, 0.943)
	**0.25**	0.959 (0.940, 0.977)	0.960 (0.939, 0.978)	0.957 (0.928, 0.979)	0.949 (0.930, 0.967)	0.962 (0.940, 0.980)
	**0.50**	0.967 (0.943, 0.986)	0.977 (0.964, 0.988)	0.969 (0.949, 0.984)	0.934 (0.901, 0.961)	0.943 (0.915, 0.968)
	**1.00**	0.947 (0.884, 0.988)	0.954 (0.921, 0.983)	0.976 (0.966, 0.984)	0.964 (0.943, 0.982)	0.955 (0.933, 0.976)
		AUPR Results on External Test Set				
		**Echo-CLR**	**Kinetics-400**	**Random**	**MI-SimCLR**	**SimCLR**
**Train Ratio**	**0.01**	0.066 (0.040, 0.121)	0.023 (0.013, 0.048)	0.008 (0.007, 0.010)	0.082 (0.018, 0.189)	0.011 (0.008, 0.016)
	**0.05**	0.194 (0.113, 0.355)	0.095 (0.049, 0.169)	0.025 (0.012, 0.062)	0.245 (0.116, 0.421)	0.016 (0.011, 0.025)
	**0.10**	0.190 (0.113, 0.312)	0.116 (0.042, 0.232)	0.144 (0.075, 0.286)	0.243 (0.112, 0.413)	0.123 (0.062, 0.267)
	**0.25**	0.217 (0.120, 0.356)	0.165 (0.109, 0.303)	0.275 (0.153, 0.480)	0.137 (0.084, 0.238)	0.294 (0.157, 0.471)
	**0.50**	0.420 (0.254, 0.597)	0.342 (0.201, 0.519)	0.270 (0.154, 0.430)	0.124 (0.074, 0.218)	0.233 (0.107, 0.383)
	**1.00**	0.337 (0.210, 0.546)	0.340 (0.187, 0.527)	0.238 (0.152, 0.399)	0.296 (0.171, 0.498)	0.394 (0.228, 0.573)

Severe AS classification results, as measured by AUROC and AUPR, for all fine-tuning ratios on both the internal and external test sets. “Train ratio” refers to the proportion of the available training data used for fine-tuning after initializing the model with the method specific by each column title. Values in parentheses represent 95% confidence intervals determined by bootstrapping the test set. AS = aortic stenosis; AUPR = are under the precision-recall curve; AUROC = area under the receiver operating characteristic curve; MI-SimCLR = multi-instance SimCLR.

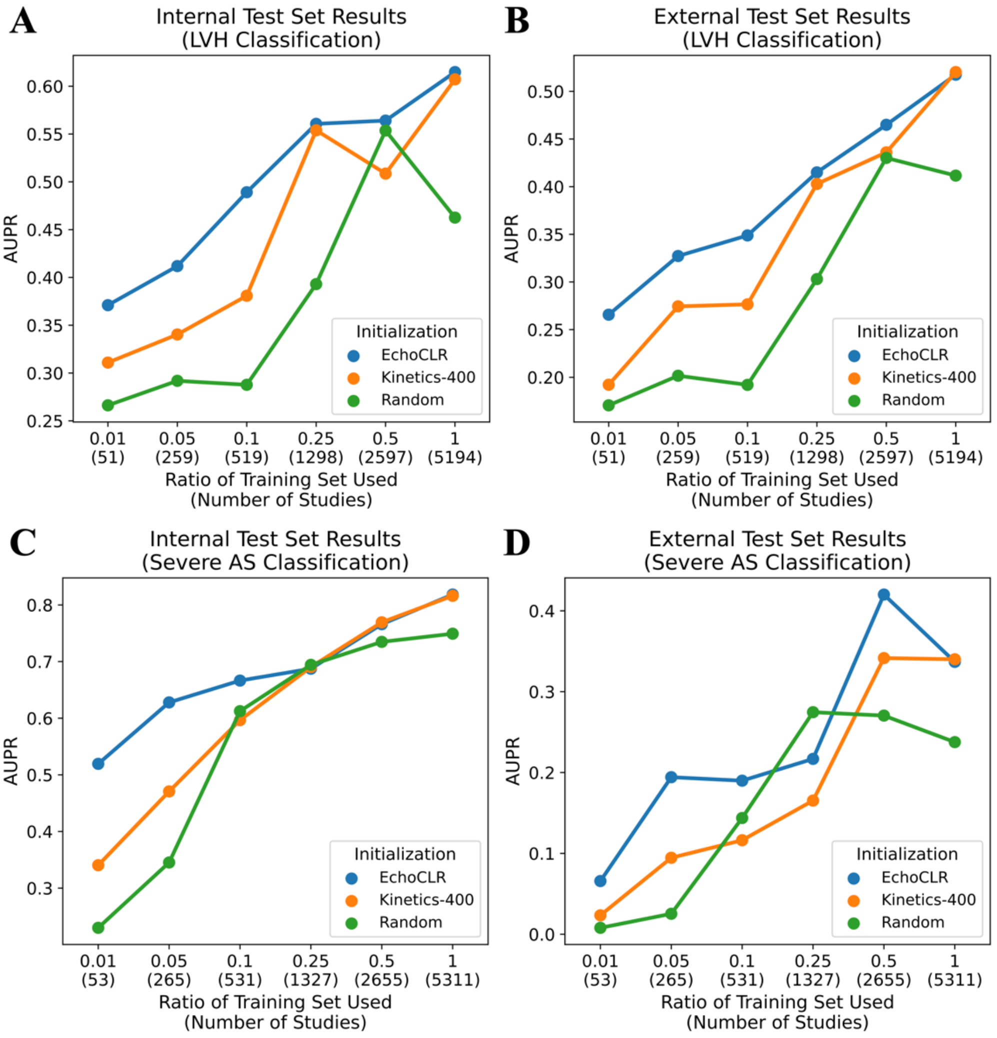

Fig. S1 | Comparing contrastive learning methods when fine-tuned on different amounts of training data. AUPR for LVH classification on the internal **(A)** and external test set **(B)** and severe AS classification on the internal **(C)** and external test set **(D)** for a randomly initialized, Kinetics-400-pretrained, and EchoCLR-pretrained model when fine-tuned on different amounts of labeled training data. AS = aortic stenosis; AUPR = area under the precision-recall curve; LVH = left ventricular hypertrophy.

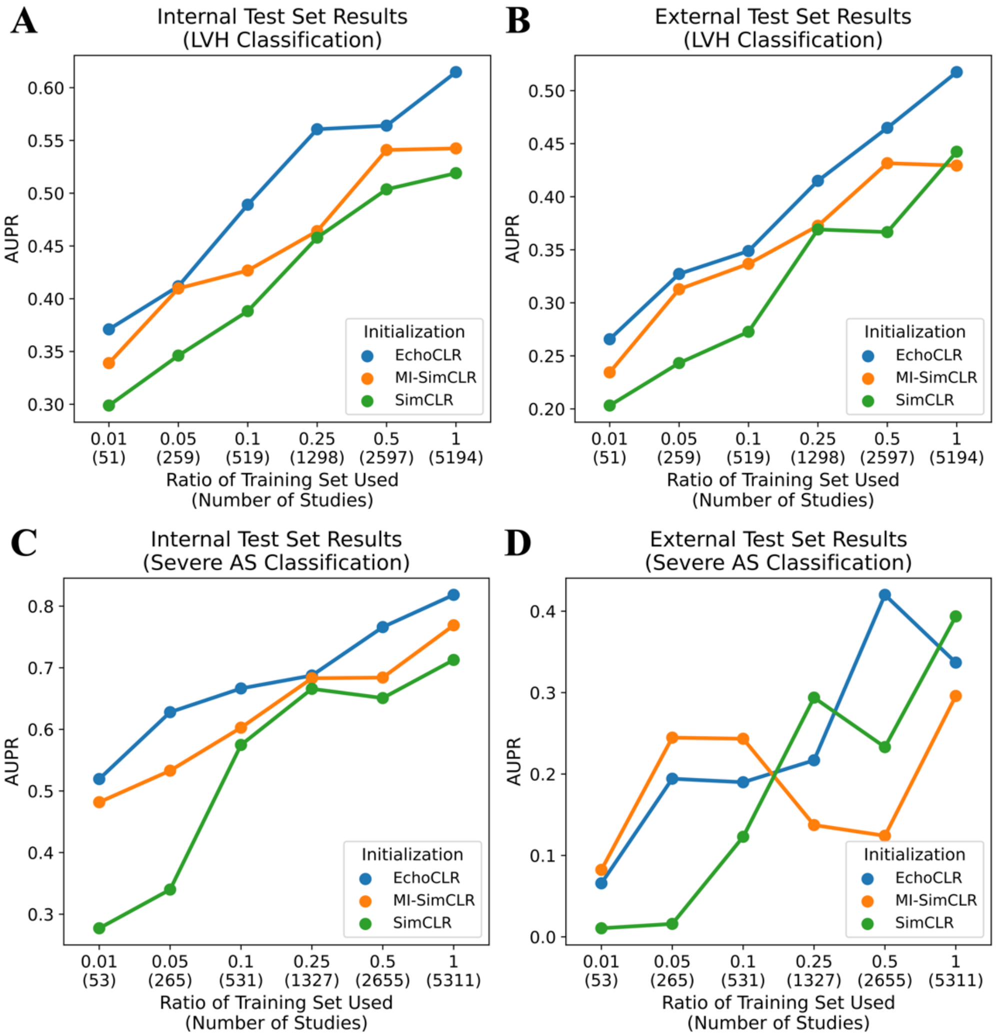

Fig. S2 | Additional ablation study of EchoCLR when finetuned on different amounts of training data. AUPR for LVH classification on the internal **(A)** and external test set **(B)** and severe AS classification on the internal **(C)** and external test set **(D)** for a SimCLR-pretrained, MI-SimCLR-pretrained, and EchoCLR-pretrained model when fine-tuned on different amounts of labeled training data. AS = aortic stenosis; AUPR = area under the precision-recall curve; LVH = left ventricular hypertrophy; MI-SimCLR = multi-instance SimCLR.
