# Supplementary material for: Efficient deep learning-based automated diagnosis from echocardiography with contrastive self-supervised learning
Source: Commun Med (Lond). 2024 Jul 6;4:133. doi: 10.1038/s43856-024-00538-3 (PMC11227494; doi:10.1038/s43856-024-00538-3)
Supplement: Supplementary file 2 — Description of Additional Supplementary Files [file 43856_2024_538_MOESM2_ESM.pdf]

Description of Additional Supplementary Files

File name: Supplementary Data 1

Description: Data used to create Figures 2 and 3.
