# Supplementary material for: Efficient deep learning-based automated diagnosis from echocardiography with contrastive self-supervised learning
Source: Commun Med (Lond). 2024 Jul 6;4:133. doi: 10.1038/s43856-024-00538-3 (PMC11227494; doi:10.1038/s43856-024-00538-3)
Supplement: Supplementary file 4 — Reporting Summary [file 43856_2024_538_MOESM4_ESM.pdf]

nature portfolio

Corresponding author(s): Rohan Khera

Last updated by author(s): Oct 19, 2023

Reporting Summary

Nature Portfolio wishes to improve the reproducibility of the work that we publish. This form provides structure for consistency and transparency in reporting. For further information on Nature Portfolio policies, see our Editorial Policies and the Editorial Policy Checklist.

Statistics

For all statistical analyses, confirm that the following items are present in the figure legend, table legend, main text, or Methods section.

n/a	Confirmed	
☐	☒	The exact sample size (*n*) for each experimental group/condition, given as a discrete number and unit of measurement
☒	☐	A statement on whether measurements were taken from distinct samples or whether the same sample was measured repeatedly
☐	☒	The statistical test(s) used AND whether they are one- or two-sided<br>*Only common tests should be described solely by name; describe more complex techniques in the Methods section.*
☐	☒	A description of all covariates tested
☒	☐	A description of any assumptions or corrections, such as tests of normality and adjustment for multiple comparisons
☐	☒	A full description of the statistical parameters including central tendency (e.g. means) or other basic estimates (e.g. regression coefficient) AND variation (e.g. standard deviation) or associated estimates of uncertainty (e.g. confidence intervals)
☒	☐	For null hypothesis testing, the test statistic (e.g. *F*, *t*, *r*) with confidence intervals, effect sizes, degrees of freedom and *P* value noted<br>*Give P values as exact values whenever suitable.*
☒	☐	For Bayesian analysis, information on the choice of priors and Markov chain Monte Carlo settings
☒	☐	For hierarchical and complex designs, identification of the appropriate level for tests and full reporting of outcomes
☒	☐	Estimates of effect sizes (e.g. Cohen's *d*, Pearson's *r*), indicating how they were calculated

Our web collection on statistics for biologists contains articles on many of the points above.

Software and code

Policy information about availability of computer code

Data collection	Imaging data was extracted and preprocessed with the pydicom (2.2.2), numpy (1.20.3), and opencv-python (4.5.4.58) packages in Python. Full details and dependencies can be found at https://github.com/CarDS-Yale/EchoCLR/blob/main/echo.yml.
Data analysis	Deep learning model development and analysis was performed with Python packages including numpy (1.20.3), opencv-python (4.5.4.58), pytorch (1.8.0), torchvision (0.9.0), pandas (1.3.4), matplotlib (3.4.3), seaborn (0.11.2), scikit-learn (1.0.2), and scipy (1.7.3). All code is publicly available at https://github.com/CarDS-Yale/EchoCLR.

For manuscripts utilizing custom algorithms or software that are central to the research but not yet described in published literature, software must be made available to editors and reviewers. We strongly encourage code deposition in a community repository (e.g. GitHub). See the Nature Portfolio guidelines for submitting code & software for further information.

Data

Policy information about availability of data

All manuscripts must include a data availability statement. This statement should provide the following information, where applicable:

- Accession codes, unique identifiers, or web links for publicly available datasets
- A description of any restrictions on data availability
- For clinical datasets or third party data, please ensure that the statement adheres to our policy

The data used in this study are not currently available for public sharing given the restrictions in our institutional review board approval. Deidentified test data may be made available to researchers under a data use agreement after publication in a peer-reviewed journal.

Research involving human participants, their data, or biological material

Policy information about studies with human participants or human data. See also policy information about sex, gender (identity/presentation), and sexual orientation and race, ethnicity and racism.

Reporting on sex and gender	Self-reported gender was determined from the electronic health record. Gender-based analysis was not performed since this study focused on the methodological development of an algorithm and its efficiency. However, we included a representative cohort that was 49% female and 51% male.
Reporting on race, ethnicity, or other socially relevant groupings	Self-reported race was determined from the electronic health record. Race-reported analysis was not performed since this study focused on the methodological development of an algorithm and its efficiency.
Population characteristics	Detailed population characteristics can be found in Table S1.
Recruitment	Participants were not recruited. This study utilized retrospective echocardiographic imaging data from routine patient care at Yale New Haven Health System hospitals from 2016-2021.
Ethics oversight	This study was approved by the Yale Institutional Review Board (IRB), which waived the need for informed consent since the study represents secondary analysis of existing data.

Note that full information on the approval of the study protocol must also be provided in the manuscript.

Field-specific reporting

Please select the one below that is the best fit for your research. If you are not sure, read the appropriate sections before making your selection.

☒ Life sciences ☐ Behavioural & social sciences ☐ Ecological, evolutionary & environmental sciences

For a reference copy of the document with all sections, see nature.com/documents/nr-reporting-summary-flat.pdf

Life sciences study design

All studies must disclose on these points even when the disclosure is negative.

Sample size	Sample size calculations were not performed. Deep learning models were trained on various amounts of labeled data in order to assess and compare "label efficiency" (how predictive performance scales with the amount of training data).
Data exclusions	Echocardiogram videos from all views other than parasternal long axis (PLAX) were excluded, as determined by an automated view classifier, since PLAX is the most commonly acquired view for diagnosis of aortic stenosis (AS). Low-flow, low-gradient "paradoxical" AS cases, as determined by the final clinical report, were excluded since they do not represent typical AS presentation. These exclusion criteria were determined before the study was carried out.
Replication	All predictive performance measures of deep learning models are presented with 95% confidence intervals obtained by 10,000 bootstrap samples of the evaluation set.
Randomization	Echocardiographic studies from the 2016-2020 cohort were randomly partitioned into training (75%), validation (10%), and internal test (15%) sets. Data from 2021 was used as a temporally distinct external test set.
Blinding	Blinding was not applicable to this study, which evaluated predictive performance of automated deep learning systems.

Reporting for specific materials, systems and methods

We require information from authors about some types of materials, experimental systems and methods used in many studies. Here, indicate whether each material, system or method listed is relevant to your study. If you are not sure if a list item applies to your research, read the appropriate section before selecting a response.

Materials & experimental systems

n/a	Involved in the study
☒	☐ Antibodies
☒	☐ Eukaryotic cell lines
☒	☐ Palaeontology and archaeology
☒	☐ Animals and other organisms
☒	☐ Clinical data
☒	☐ Dual use research of concern
☒	☐ Plants

Methods

n/a	Involved in the study
☒	☐ ChIP-seq
☒	☐ Flow cytometry
☒	☐ MRI-based neuroimaging

Plants

Seed stocks

Report on the source of all seed stocks or other plant material used. If applicable, state the seed stock centre and catalogue number. If plant specimens were collected from the field, describe the collection location, date and sampling procedures.

Novel plant genotypes

Describe the methods by which all novel plant genotypes were produced. This includes those generated by transgenic approaches, gene editing, chemical/radiation-based mutagenesis and hybridization. For transgenic lines, describe the transformation method, the number of independent lines analyzed and the generation upon which experiments were performed. For gene-edited lines, describe the editor used, the endogenous sequence targeted for editing, the targeting guide RNA sequence (if applicable) and how the editor was applied.

Authentication

Describe any authentication procedures for each seed stock used or novel genotype generated. Describe any experiments used to assess the effect of a mutation and, where applicable, how potential secondary effects (e.g. second site T-DNA insertions, mosiacism, off-target gene editing) were examined.
